# The heart of Detroit study: a window into urban middle-aged and older African Americans’ daily lives to understand psychosocial determinants of cardiovascular disease risk

**DOI:** 10.1186/s12888-023-05148-2

**Published:** 2023-10-18

**Authors:** Kristin M. Davis, Katherine Knauft, Lena Lewis, Michael Petriello, Lauren Petrick, Francesca Luca, Nataria T. Joseph, Heather Fritz, Malcolm Cutchin, Lance Rappaport, Phillip Levy, Christopher G. Engeland, Samuele Zilioli

**Affiliations:** 1 Department of Psychology, Wayne State University, 5057 Woodward Avenue, Detroit, MI 48202 USA; 2 College of Human Medicine, Michigan State University, East Lansing, MI 48824 USA; 3 Institute of Environmental Health Sciences, Department of Pharmacology, Wayne State University, Detroit, MI 48201 USA; 4 Department of Environmental Medicine and Public Health, Icahn School of Medicine at Mount Sinai, New York, NY 10029 USA; 5 Center for Molecular Medicine and Genetics, Wayne State University, Detroit, MI 48201 USA; 6 Department of Obstetrics and Gynecology, Wayne State University, Detroit, MI 48201 USA; 7 Department of Psychology, Pepperdine University, Malibu, CA 90265 USA; 8 School of Occupational Therapy, Pacific Northwest University of Health Sciences, Yakima, WA 98901 USA; 9 Department of Psychology, University of Windsor, Windsor, ON N9B 1B4 Canada; 10 Departments of Emergency Medicine and Physiology, Wayne State University, Detroit, MI 48201 USA; 11 Department of Biobehavioral Health, Pennsylvania State University, University Park, PA 16802 USA; 12 Ross and Carol Nese College of Nursing, Pennsylvania State University, University Park, PA 16802 USA; 13 Department of Family Medicine and Public Health Sciences, Wayne State University, Detroit, MI 48201 USA

**Keywords:** Stress, Racism, African Americans, Cardiovascular Disease, Inflammation, Ecologically momentary Assessment, Mixed-Method Approach, Cortisol

## Abstract

**Background:**

Cardiovascular disease disproportionately affects African Americans. Psychosocial factors, including the experience of and emotional reactivity to racism and interpersonal stressors, contribute to the etiology and progression of cardiovascular disease through effects on health behaviors, stress-responsive neuroendocrine axes, and immune processes. The full pathway and complexities of these associations remain underexamined in African Americans. The Heart of Detroit Study aims to identify and model the biopsychosocial pathways that influence cardiovascular disease risk in a sample of urban middle-aged and older African American adults.

**Methods:**

The proposed sample will be composed of 500 African American adults between the ages of 55 and 75 from the Detroit urban area. This longitudinal study will consist of two waves of data collection, two years apart. Biomarkers of stress, inflammation, and cardiovascular surrogate endpoints (i.e., heart rate variability and blood pressure) will be collected at each wave. Ecological momentary assessments will characterize momentary and daily experiences of stress, affect, and health behaviors during the first wave. A proposed subsample of 60 individuals will also complete an in-depth qualitative interview to contextualize quantitative results. The central hypothesis of this project is that interpersonal stressors predict poor cardiovascular outcomes, cumulative physiological stress, poor sleep, and inflammation by altering daily affect, daily health behaviors, and daily physiological stress.

**Discussion:**

This study will provide insight into the biopsychosocial pathways through which experiences of stress and discrimination increase cardiovascular disease risk over micro and macro time scales among urban African American adults. Its discoveries will guide the design of future contextualized, time-sensitive, and culturally tailored behavioral interventions to reduce racial disparities in cardiovascular disease risk.

## Background

Cardiovascular disease (CVD) has been the leading cause of death in the United States (US) for over 100 years [1, 2]. Intensive scientific efforts resulted in decades of steady decline in CVD morbidity and mortality rates; however, this improvement has stalled, and CVD rates have been stagnant in the US since 2010 [3, 4]. Racial disparities in CVD-related outcomes have remained a consistent problem [5–9]. In a study of the earliest nationally representative data on CVD mortality, from 1920 to 1955, rates in non-white Americans were “consistently higher” than in white Americans [8]. Such racial disparities have generally either remained unchanged or grown worse over time [10–12]. Current mortality rates for heart disease and stroke are 30% and 45% higher, respectively, among African Americans (AAs) compared to white Americans [13]. The scientific community has repeatedly called for action to examine and reduce racial CVD disparities [6, 13], yet these inequities are projected to deepen in coming years [14]. Overall CVD morbidity and mortality rates in the US are projected to rise through 2060, despite concurrent declines in CVD among white Americans; without meaningful intervention, racially minoritized groups will grow more disproportionately affected [14]. To reduce these disparities and reverse the recent stagnation in improvement of CVD rates, research must go beyond traditional CVD risk factors to explore the fundamental psychological, social, and environmental factors at play.

While many factors contribute to racial CVD disparities, racism may be particularly influential. Multiple theories and conceptual models converge to suggest that racism directly contributes to health disparities. Racism is a complex concept [15] and can be conceptualized as a set of beliefs, attitudes, structures, and actions that maintain systems of power and oppression along socially-defined racial lines [16, 17]. Three interrelated sources of racism have been delineated: structural, cultural, and individual [16, 18]. The Multidimensional Model of Racism-Related Stress [16] posits that these sources of racism are enacted across various contexts, including the interpersonal (e.g., interactions between individuals) and the sociopolitical (e.g., policy choices). Racism enacted across these sources and contexts results in the racism-related stress that is uniquely experienced by racially minoritized groups [16] and contributes to racial health disparities [17].

The interpersonal context is particularly important in the association between racism-related stress and racial health disparities. Interpersonal racism manifests as acts of prejudice and discrimination toward individuals. According to the Biopsychosocial Model of Racism [19], experiences of prejudice or discrimination are powerful stressors with psychological, behavioral, and biological sequelae. Repeated stressful experiences lead to chronic activation of the stress response system, resulting in the ‘wear and tear’ on the body (i.e., allostatic load [20] that has been found to partially explain black-white disparities in premature mortality [21]. Dysregulation of the hypothalamic-pituitary-adrenal (HPA) axis (e.g., flattened diurnal slopes, glucocorticoid resistance) and elevated systemic inflammation are critical components of the allostatic load model, and both contribute to CVD risk [22–27]. Past research has found that experiences of discrimination are associated with flatter diurnal cortisol slopes [28] and increased systemic inflammation [29, 30]. Furthermore, discrimination has been directly linked to increases in hypertension, carotid intima-media thickness, and incident of CVD [31–33]. Additionally, behavioral reactions to chronic stress (e.g., poor sleep quality) can also link experiences of stress and discrimination to poorer cardiovascular outcomes, possibly via pro-inflammatory mechanisms [34–36]. Thus, experiences of discrimination have psychological, biological, and behavioral consequences that negatively affect long-term health and contribute to racial health disparities.

The Biopsychosocial Model of Racism also recognizes that experiences of discrimination need to be considered in the context of other social determinants of health, such as socioeconomic status (SES) and interpersonal relationships. The association between low SES and increased CVD risk can be attributed to many factors [37], including increased inflammation and abdominal adiposity [38], elevated blood pressure [39, 40], increased tobacco use [41], and reduced psychosocial resources [42, 43]. Financial strain, a stressor associated with low SES, has been linked to experiencing more negative social interactions [44]. Patterns of negative social interactions have been found to be relatively stable over time, thus serving as a source of chronic stress [45, 49]. Among older adults, social and family networks can offer extensive support but can also be sources of interpersonal conflict [46, 47]. In turn, frequent negative interaction can negatively influence both physical and mental health [44, 48, 49]. For example, negative social interactions have been associated with increased carotid artery intima-medial thickness [50] and incident hypertension [48]. Loneliness and social isolation are similarly associated with increased CVD risk [51], possibly via pro-inflammatory mechanisms [52, 53]. Lastly, while higher SES is generally associated with lower CVD risk [37, 54], studies have found that racial disparities in CVD risk factors, such as hypertension, high cholesterol, obesity, diabetes, and metabolic syndrome remain constant or increase with increasing SES [55–57]. Higher SES may therefore not confer the same health benefits to AAs, likely due to the added burden of structural and interpersonal racism [55, 58].

The Intersectionality Theory [59] and more specifically the concepts of gendered racism [60] highlight the importance of considering gender-specific forms of discrimination. Intersectionality Theory recognizes that all individuals are members of multiple social groups simultaneously (e.g., racial groups, gender groups), and examining the effect of membership in any one social group in isolation is thus inherently limited [59]. Membership in multiple subordinated groups can produce additive or multiplicative effects on health. The intersection of race and gender has been a focal point of intersectionality theory since its inception [59, 61]. For example, Black women may experience “double discrimination”, that is the combined effects of discrimination based on both race and gender [61], and previous studies have shown that experiences of interpersonal gendered racism negatively affect self-reported mental and physical health in Black women [62]. Structural gendered racism, that is gendered racism that operates at the institutional and cultural level, also significantly contributes to racial disparities in health, including maternal mortality [63] and increased risk for COVID-19 [64]. Overall, the intersecting effects of gendered racism on health remain understudied.

These three theoretical models – the Multidimensional Model of Racism-Related Stress, the Biopsychosocial Model of Racism, and Intersectionality Theory – provide a framework for much-needed research examining the pathways through which racism and other psychosocial determinants of health impact CVD risk among AAs. Based on this theoretical foundation, The Heart of Detroit Study was designed to identify and conceptualize, through a mixed-method approach, the main psychosocial stressors most salient for urban middle-aged and older AAs and model the daily psychological, behavioral, and biological pathways through which these factors may exacerbate CVD risk in this population.

## Methods and study design

### Project overview

The Heart of Detroit Study is an intensive longitudinal mixed-method study that combines Ecological Momentary Assessment (EMA), biological, and qualitative approaches. The sample consists of self-identified AA participants between the ages of 55 and 75 living in the Tri-County area of Metro Detroit. The Heart of Detroit Study is a prospective longitudinal study in which participants are assessed extensively using a multimodal design at baseline (Wave 1) then followed up 2 years later (Wave 2). Wave 1 consists of two home visits, separated by a seven-day period of EMA data collection. A subset of participants will be asked to participate in an additional semi-structured qualitative interview. Wave 2 of quantitative data collection will take place two years after the Wave 1 baseline and consists of one home visit.

### Participants

#### Inclusion and exclusion criteria

Individuals who self-identify as AA, are between the ages of 55 and 75 years, and are able to read and write in English are potentially eligible to participate in Wave 1. The main exclusion criteria include a history of CVD at baseline assessment, HIV/AIDS infection, neurodegenerative disease, psychiatric disorders, or cognitive impairment, and having plans to move out of the study area within the next two years. The proposed sample size is 500 individuals.

#### Recruitment and enrollment

Participants are being recruited through the Wayne State Institute of Gerontology Participant Research Pool, a pool of AAs living in Metro Detroit who are willing to participate in research [65]. Other recruitment methods include online advertisements, advertisements placed in the community, and snowball sampling. All potential participants complete an initial phone screening after which eligible individuals are scheduled for their first home visit.

### Procedures

Study procedures were approved by the Wayne State University Institutional Review Board. During the first home visit, participants provide written informed consent, and eligibility is confirmed. Following the consent and eligibility procedures, participants complete questionnaires about demographic information, physical and psychological health, health behaviors, cognitive abilities, and personal life experiences. For the next seven consecutive days, participants self-assess blood pressure each morning and evening using a provided home blood pressure monitor, provide four saliva samples per day at specified times, and record information about these measures in a daily log. Additionally, participants are asked to wear an actigraphy watch and carry a cell phone to complete EMA measures from awakening until bedtime. Both the actigraphy watch and the cell phone are provided by the study and are considered minimally invasive. Following the daily monitoring period, a second home visit is conducted. At the second home visit, height, weight, waist and hip circumference, peak expiratory flow, heart rate variability, and blood pressure are measured by study staff; hair and venous blood samples are also collected. Participants also complete questionnaires about health behaviors at this visit. A proposed subsample of 100 participants will provide capillary blood microsamples from a finger-prick. Blood microsamples are collected by research staff; however, these devices can also be used for personal, home-based collection. Blood volumes collected through blood microsamples are adequate for most clinically-relevant biomarkers including those related to the environment, inflammation, and nutrition even in times of decreased access to healthcare [66]. Two years after Wave 1, Wave 2 will be conducted. Wave 2 consists of a single home visit, in which participants will be asked to complete demographic, health, behavioral, cognitive, psychological, and experiential questionnaires. During this visit, study staff will collect many of the measures and questionnaires collected at Wave 1, including hair and blood samples. A subset of participants will be asked to complete a semi-structured qualitative interview intended to contextualize and help explain the quantitative findings. Participants will be compensated up to $460 for their time and participation in the study.

### Measures

#### Background and anthropometric variables

Demographic information (e.g., age, sex, and SES) are assessed once per wave during home visits. Measures of neighborhood quality are obtained via questionnaire; census block group data will be used to generate a Neighborhood Adversity Index and Residential Segregation measure that have been linked to stress and health outcomes [67, 68]. Other background information is also collected, including measures of personality traits, cognitive function, and mental health. Anthropometric variables, including height, weight, and waist circumference, are measured by study staff.

#### Stress and discrimination

Stress is a multifaceted process comprised of several domains that occur across various timescales [69–71]. To leverage the multimodal study design and obtain a more nuanced understanding of the sources, mediators, and consequences of stress in urban AAs, several measures of stress are being used.

Global measures of stress are assessed once per wave; these measures capture chronic stressors and major life event stressors. Chronic stressors assessed include neighborhood adversity [72], everyday experiences of discrimination [73], loneliness [74], and caregiving [75]. To enable a closer examination of questions related to intersectionality, gender-specific questionnaires about experiences of discrimination and exposure to stereotypes are also used, such as the Gendered Racial Microaggressions scale [76] and the African American Men’s Gendered Racism Stress Inventory [77]. Major life events are infrequent but dramatic stressors, including major experiences of discrimination such as being unfairly fired or denied a promotion [78].

Examination of momentary (within-day) and daily (within-person) stressors and their impact on long-term health is a central goal of The Heart of Detroit Study. To assess within-person variability in these stressors, EMA questions are asked four times per day (9 a.m., 1 p.m., 5 p.m., and 9 p.m.) across seven consecutive days. These questions focus on current mood, recent (i.e., since the last EMA prompt) stressful experiences such as negative social interactions or experiences of discrimination, social context (e.g., engagement in social activities or interactions), and stress-related health behaviors such as smoking.

Lastly, cortisol is being used to assess the physiological impact of stress. Dysregulation of the HPA axis, which can be reflected in certain patterns of diurnal cortisol secretion, is an important mechanism through which stress may negatively affect health [22, 43, 79, 80]. While salivary cortisol enables analysis of individual differences in daily cortisol levels and day-to-day variability in HPA axis activation, hair cortisol levels are a complementary measure of systemic cortisol exposure across longer time scales [81]. Examination of both salivary and hair cortisol in The Heart of Detroit Study will allow for the examination of physiological stress across multiple time scales. During the daily diary period of Wave 1, participants collect four saliva samples per day (immediately at waking, 30 min after waking, prior to dinnertime, and at bedtime), enabling assessment of diurnal cortisol patterns. Compliance with sampling times is assessed by a microchip that records openings of each sampling vial (MEMS trackcap; Aardex, Denver, CO). Study staff collect one hair sample per wave for cortisol analysis during home visits. Cortisol concentrations will be quantified with a commercially available luminescence immunoassay (IBL International, Hamburg, Germany) at the Dresden LabService (Dresden, Germany).

### Emotional and behavioral mediators

Coping mechanisms are important mediators in the stress-health association [82]; consequently, several measures of coping styles and strategies are assessed, including measures of religious coping. Emotional reactivity to stress, such as increased negative affect, may also mediate the association between stressful experiences and health outcomes [83, 84] and is assessed during both waves. Access to and use of quality social support also appears to be important in the stress-health relationship [85], while loneliness and social isolation have been linked to increased CVD risk [51, 86]. Consequently, several measures of social support [87], social isolation [88], and loneliness [74] are included. Changes in health behaviors, such as increased alcohol use or reduced sleep quality, are considered key mechanisms linking stress to poor health [89]; health behaviors, such as alcohol consumption [90], tobacco use [90], and sleep habits [91] are therefore assessed. Given the overarching goal of examining mediators between stress and health across varying time scales, emotional and behavioral mediators of stress are measured using both global questionnaires assessing habitual health behaviors and EMA questions. Sleep is assessed both subjectively via questionnaire and daily diaries, and objectively using actigraphy data.

### Markers of CVD risk

Heart rate variability (HRV) is a noninvasive marker of autonomic nervous system activation [92] that is associated with both CVD risk and risk factors [93, 94] and may be a mediator between stress and CVD risk [95]. HRV is measured once per wave by study staff using a Polar v800 Heart Rate Monitor (or Polar Watch) and respiration band. After being fitted with the monitor and respiratory band, participants are asked to rest in a seated position while heart rate variability is measured for 5 consecutive minutes.

High blood pressure has the strongest evidence for CVD causation of any traditional risk factor [96], and there are marked racial disparities in the prevalence and treatment of high blood pressure [97]. While single measures of blood pressure strongly predict CVD risk, within-person variability in blood pressure predicts CVD risk above and beyond mean blood pressure [98]. Thus, two approaches to blood pressure measurement are used in The Heart of Detroit Study. First, participants self-assess blood pressure each day of the seven-day daily monitoring period using an automated device (Omron BP710N) twice per day, after awakening in the morning and before bed at night. At each time point, blood pressure is measured in triplicate, with a two-minute rest between measures. The average of these three readings is provided by the device. Participants are asked to record the time and results of each assessment. Additionally, study staff assess participants’ blood pressure once per wave using an automated device (Omron 907XL), which is considered the gold standard for office-based measurement [99]. Following the HRV measurement at each assessment, blood pressure is measured three times using an automated protocol which includes a two-minute rest between readings; the average of these three readings is taken.

Immune dysregulation and chronic inflammation are key mediators linking stress to CVD [89, 100]. Evidence links experiences of discrimination to increased inflammation; however, additional research using a greater variety of immune markers is needed to better characterize this association [29]. Consequently, The Heart of Detroit Study assesses a range of immune-related biomarkers derived from blood samples, including C-Reactive Protein (CRP), circulating inflammatory cytokines, and ex vivo lipopolysaccharide-stimulated inflammatory cytokine production. Both pro-inflammatory (e.g., IL-6) and anti-inflammatory cytokines (e.g., IL-10) are measured under basal and stimulated conditions. For basal cytokines, a portion of each blood sample is centrifuged (15 min x 1500 g), and 400 µl aliquots of the resulting supernatant are stored in a − 80^o^C freezer. For ex vivo stimulated cytokine production, 1 ml of whole blood from each sample is combined with a 3 ml solution of lipopolysaccharide (LPS; E. coli, Sigma) to create a 1 µg/ml LPS solution, and then incubated at 37 °C in 5% CO2 for 4 h on a rotational shaker. The samples are then centrifuged (15 min x 1500 g), aliquoted into 500 µl samples, and stored at -80^o^C. Plasma samples are then analyzed in Dr. Christopher Engeland’s Stress and Immunity laboratory at the Pennsylvania State University using V-Plex multiplex assays (Meso Scale Diagnostics, Rockville MD) following the manufacturer protocol. Other markers of immune function, such as white blood cell counts, will also be measured at the Detroit Medical Center. In addition to immune-related factors, blood samples will be also used to assess lipids, including total cholesterol and low-density lipoprotein [LDL] cholesterol, using Beckman Coulter Clinical Chemistry analyzers at the Detroit Medical Center. Additional blood is also being collected for future genetic, epigenetic, and gene expression analyses.

#### Qualitative interview

Following partial completion of Wave 1, a subsample of participants (proposed N = 60) will be recruited to take part in semi-structured qualitative interviews. The subsample will be selected based on stress levels and CVD risk levels during Wave 1 (high stress/high CVD risk, high stress/low CVD risk, low stress/high CVD risk, low stress/low CVD risk). Semi-structured interviews are conducted at participants’ homes using the Lifeline Interview Method (LIM) [101] (Assink & Schroots, 2010). The LIM adds value to a traditional semi-structured interview because charting the life-line (a) primes individuals to think about the key events in their life course, (b) provides an organizing structure to ensure a standardized, yet comprehensive approach to discussing the overall life course, and (c) provides anchoring points to discuss the prevailing conditions, perceptions, and responses tied to each event. The interview consists of the participant tracing major ups and downs in their life on a graph and marking major life events on that line. The interview questions are focused on each event asking for detail about: what happened, their appraisal (effects, feelings) of it, how they responded, outcomes of the event, conditions at the time (work, family, finances, housing/neighborhood, social relationships, roles, health), and that event relationships with other events on the line. A final question asks the participant to reflect on their overall lifeline. Efforts are made by the team and collaborators to ensure that the interview questions are culturally informed.

### Analytic plan

The Heart of Detroit Study includes collection and analysis of both quantitative and qualitative data. EMA data collected for seven consecutive days will evaluate the momentary and daily dynamics through which negative affect, health behaviors, and interpersonal stressors, namely discrimination [102, 103], social isolation [104], and negative interactions with close others [105], impact cortisol secretion, blood pressure, and sleep. Quantitative data will also be used to investigate concurrent and prospective associations of psychosocial stressors and health behaviors with biomarkers of cumulative stress, inflammation, and CVD surrogate endpoints. Qualitative data will allow us to probe more deeply into life conditions, processes, and behaviors beyond the quantitative measures to understand and begin to explain how various social and individual determinants of health affect each subgroup.

#### Quantitative data analyses

Multilevel structural equation models (MSEM) will evaluate contributions of interpersonal stress experiences (e.g., discrimination, negative social interactions) to altered cortisol secretion, elevated blood pressure, and impaired sleep at least partially through elevated negative affect and adverse health behaviors (e.g., smoking). The MSEM framework can parse within-person and between-person covariation to test whether stressful experiences within daily life contribute to increased momentary negative affect, engagement in negative health behaviors, and, subsequently, to alterations in cortisol secretion (i.e., flattened daily cortisol slopes), elevated evening blood pressure, and worsened sleep. The capacity of the MSEM framework to statistically parse within-person and between-person covariation also facilitates adjustment for any potential between-person covariation (i.e., at level 3) to simultaneously account for theoretically proposed and empirically demonstrated between-person associations among stress, negative affect, negative health behaviors, cortisol secretion, blood pressure, and sleep [82, 106–110]. Three-level models will be constructed to examine within-person and within-day associations (Level 1), within-person and between-day associations (Level 2), and between-persons associations (Level 3) among the variables of interest.

Structural equation model (SEM) and MSEM frameworks will also examine associations of baseline interpersonal stress exposure, averaged over the 7-day recording period, with adverse health outcomes, hair cortisol, inflammation, resting blood pressure, and HRV both concurrently and prospectively over the 2-year follow-up period. For example, health outcomes, hair cortisol, inflammation, resting blood pressure, and HRV at baseline and latent change from baseline to 2-year follow-up will be regressed onto latent interpersonal stress exposure at baseline. Similarly, the MSEM approach will be extended to evaluate concurrent and prospective associations of reactivity to interpersonal stress (i.e., the within-person covariation of interpersonal stress with negative affect [emotional reactivity] and negative health behaviors [behavioral reactivity]) with adverse health outcomes, hair cortisol, inflammation, resting blood pressure, and HRV.

### Qualitative data analysis

To examine the data from the semi-structured qualitative interviews, Grounded Theory Analysis (GTA) will be applied to the interview transcripts and field notes. In the GTA method, key conceptual categories are constructed from a dataset to theoretically explain the most important processes that emerge from the data [111]. Following established GTA procedures, coders will be sensitized to examine the conditions, contexts, actions, and consequences present in the text. Codes will be progressively honed and aggregated via a constant comparative analysis to develop core conceptual categories. Conceptual categories will then be connected in a way that explains how conditions, dynamics, and behaviors (for instance) are related to stress and CVD risk.

### Sample size calculation

To calculate the sample size required for the EMA component, power analyses based on Monte Carlo simulation were conducted with 2,000 independent trials. While uncommon in multilevel models, pseudo-standardized regression paths at levels 1 and 2 (i.e., b) were estimated by simulating associated variances at 1 given little prior research to guide the a priori determination of variance estimates. A sample of 500 people, with 35 assessments over 7 days, provides > 80% statistical power to detect (1) small associations of momentary interpersonal stress with current negative affect or negative health behaviors (b = 0.03), and (2) small associations of daily interpersonal stress with negative affect, negative health behaviors, elevated blood pressure, or altered cortisol secretion (b = 0.08).

To calculate the sample size required for CVD-related outcomes, power analyses were based on two-tailed significance tests using an α of 0.05 and are reported in an effect size *r* metric (r = sqrt(t2/t2 + df); [112]), which is computed from t values associated with betas obtained from MSEM. A sample of 500 individuals provide > 80% statistical power to evaluate small and medium effects (|*r*|= 0.14).

#### Missing data and model assumptions

All SEM and MSEM analyses will use full information maximum likelihood (FIML) estimation with a sandwich estimator robust to deviations from multivariate normality [113–115] and data missing at random conditional on modeled covariates [116, 117]. Additional techniques to account for missing data will be considered, including multiple imputations using chained equations (MICE) [118] and inverse probability weighting to calibrate the data [119, 120].

## Discussion

The Heart of Detroit Study is a multi-faceted project that seeks to attain a more comprehensive understanding of the relationship between psychosocial stress and CVD risk in urban AA middle-aged and older adults. The study design allows for examination of links between short-term processes (i.e., momentary and daily stress) and cardiovascular health trajectories across multiple timespans (moments, days, years), methodological approaches (quantitative, qualitative), and biobehavioral systems (e.g., subjective and objective health behaviors; HPA axis, immune system, cardiovascular system functioning). The inclusion of EMA and daily diary measures enables a fine-grained and ecologically valid examination of interpersonal stressors, such as experiences of discrimination, and associated emotional and behavioral responses in this population. Including both EMA and daily diary approaches allows for better characterization of the contextualized, event-dependent links between psychosocial stressors and CVD risk on a within-person level, facilitating the development of more targeted and effective interventions. By including multiple temporal scales (momentary assessment, daily assessment, and two-year follow-up), this study is well-suited to examine the pathways between both moment-to-moment and day-to-day variability in individuals’ experiences and long-term change in their cardiovascular health. Finally, the qualitative portion of our mixed-methods approach will supplement and contextualize quantitative findings, providing insight into participants’ experiences of and responses to the stressors in their daily lives.

The Heart of Detroit Study will provide valuable insights into the direct and indirect associations between stress and CVD risk in AAs through the incorporation of both traditional (e.g., blood lipids) and novel (e.g., stimulated pro-inflammatory cytokine production) biomarkers of CVD risk. Examination of these biomarkers will allow for examination of the role of multiple biological pathways through which interpersonal stressors may influence health across time. The use of EMA, daily diaries, and in-depth qualitative interviews combined with a detailed biobehavioral characterization of participants’ CVD risk will help contextualize previously identified psychosocial stressors and behaviors among AAs and identify the optimal timing for interventions to mitigate subclinical CVD progression. These results will help inform contextualized, culturally tailored, and adaptive behavioral interventions aimed at reducing racial disparities in CVD, an area of research that remains underdeveloped.

While the primary focus of The Heart of Detroit Study is on the health effects of experiences of interpersonal stressors, including interpersonal racism, structural racism remains the critical determinant of racial disparities in CVD outcomes [13, 121]. For example, historical discriminatory lending and insurance policies, such as redlining, created a structural barrier that prevented racially minoritized groups from moving and resulted in racially segregated neighborhoods [122]. In addition, these policies have led to environmental injustices, manifested, for example, with racially minoritized groups being most impacted by pollution and pollutant-induced chronic diseases [123]. This race-based residential segregation remains a fundamental driver of racial health disparities: by fueling the concentration of poverty and producing inequitable access to resources at both the individual and community level, racial segregation drives racial disparities in CVD-related outcomes [13, 58, 124–126]. Detroit has consistently been one of the most segregated metropolitan areas in the US [127, 128]. Past research conducted in Detroit has found that redlining and racial segregation are important predictors of health outcomes, including poor access to health care [129], low birth weight [130], high childhood blood lead levels [131], and poor self-rated health [132], disproportionately affecting AA residents [125]. The Heart of Detroit Study includes measures of both subjective and objective neighborhood quality, with the intention of examining the impacts of these aspects of structural racism on health.

The Heart of Detroit Study focuses exclusively on AA participants. Much existing research on racial health disparities has used a between-group approach, and this research has made important contributions to our understanding of the existence and extent of such disparities. However, between-group comparisons may be insufficient to fully understand racial health disparities [133]; there are theoretical and empirical reasons for why assessments conducted strictly within the AA population will deepen scientific understanding of the health of racially minoritized groups. These reasons include the untested and unjust assumption that Whites are a standard from which AAs deviate (Cultural Deviance Model), the falsified assumptions that group differences exist only for outcomes of interest and not for underlying processes (Cultural Equivalence Model), and the importance of investigating within-group heterogeneity with appropriate statistical power [133, 134]. The exclusive focus on AAs is a strength of our study and a fruitful departure from the Cultural Deviance and Cultural Equivalence models. The Heart of Detroit Study will enable us to both address the long-standing underrepresentation of AAs in studies on daily psychosocial determinants of CVD, and appropriately capture culturally relevant dynamics and within-group heterogeneity, which would go under-examined in a multiracial version of this study. Moreover, because certain social stressors are not comparable across races, a single race study will help avoid problems related to residual confounding [135]. For example, use of a within-group design enables a more nuanced understanding of the effect of SES on the health of AAs. Importantly, socioeconomic distinctions between racial groups are multifaceted and cannot easily be summarized into individual metrics like income [135]; some of the observed racial and ethnic health disparities are therefore likely the result of unmeasured socioeconomic differences [136]. To assess SES more accurately within our sample, The Heart of Detroit Study includes a range of subjective and objective measures of SES across individual, family, and neighborhood levels. Results from The Heart of Detroit Study could therefore help disentangle the effects of race and SES on CVD risk.

## Conclusion

Racial disparities in CVD morbidity and mortality remain a pressing public health problem, with AAs at substantially higher risk. While scientific efforts have led to improvements in CVD treatment, progress in reducing racial disparities has been slow, at least in part due to insufficient research investigating the underlying social and psychological determinants of CVD risk in minoritized groups, such as AAs. Past research has been hampered by limited inclusion of AA participants, confounding by SES, and by a lack of understanding of daily stressors and subsequent behavioral and emotional responses in this population. Our results are expected to contribute to the establishment of a comprehensive model of the psychosocial determinants of CVD risk in urban AA middle-aged and older adults, which can help the development of more culturally and individually tailored treatments and interventions to reduce CVD in this population.

## Data Availability

Not applicable.

## Abbreviations

AAs: African Americans
CVD: cardiovascular disease
EMA: Ecological Momentary Assessment
FIML: full information maximum likelihood
GTA: Grounded Theory Analysis
HPA: hypothalamic-pituitary-adrenal
HRV: Heart rate variability
LDL: low-density lipoprotein
MICE: multiple imputations using chained equations
MSEM: Multilevel structural equation modeling
SEM: Structural equation modeling
SES: socioeconomic status
