# Correction: The heart of Detroit study: a window into urban middle-aged and older African Americans’ daily lives to understand psychosocial determinants of cardiovascular disease risk

**DOI:** 10.1186/s12888-023-05339-x

**Published:** 2023-11-16

**Authors:** Kristin M. Davis, Katherine Knauft, Lena Lewis, Michael Petriello, Lauren Petrick, Francesca Luca, Nataria T. Joseph, Heather Fritz, Malcolm Cutchin, Lance Rappaport, Phillip Levy, Christopher G. Engeland, Samuele Zilioli

**Affiliations:** 1 Department of Psychology, Wayne State University, 5057 Woodward Avenue, Detroit, MI 48202 USA; 2 College of Human Medicine, Michigan State University, East Lansing, MI 48824 USA; 3 Institute of Environmental Health Sciences, Department of Pharmacology, Wayne State University, Detroit, MI 48201 USA; 4 Department of Environmental Medicine and Public Health, Icahn School of Medicine at Mount Sinai, New York, NY 10029 USA; 5 Center for Molecular Medicine and Genetics, Wayne State University, Detroit, MI 48201 USA; 6 Department of Obstetrics and Gynecology, Wayne State University, Detroit, MI 48201 USA; 7 Department of Psychology, Pepperdine University, Malibu, CA 90265 USA; 8 School of Occupational Therapy, Pacific Northwest University of Health Sciences, Yakima, WA 98901 USA; 9 Department of Psychology, University of Windsor, Windsor, ON N9B 1B4 Canada; 10 Departments of Emergency Medicine and Physiology, Wayne State University, Detroit, MI 48201 USA; 11 Department of Biobehavioral Health, Pennsylvania State University, University Park, PA 16802 USA; 12 Ross and Carol Nese College of Nursing, Pennsylvania State University, University Park, PA 16802 USA; 13 Department of Family Medicine and Public Health Sciences, Wayne State University, Detroit, MI 48201 USA

**Correction: BMC Psychiatry (2023) 23:766**

10.1186/s12888-023-05148-2

Following publication of the original article [1], the authors identified an error in the author name of Phillip Levy.

The incorrect author name is: Philip Levy.

The correct author name is: Phillip Levy.

The author group has been updated above and the original article [1] has been corrected.
